# Philadelphia Hospital

**Published:** 1840-01-01

**Authors:** 


### PHILADELPHIA HOSPITAL.

### Two Cases of Rupture of the Axillary Artery, during the Reduction of Dislocations of the Humerus.

Some few years ago we presented our readers with an account of a paper, published by M. Flambert, a surgeon of, we think, the Hôtel Dieu at Lyons, in the Répertoire. In that paper the author detailed seven cases of serious injury inflicted during the reduction of dislocations of the head of the humerus. Amongst other accidents there were, we know, laceration of the brachial plexus, and of the axillary artery.

The following cases quoted from Dr. Gibson's Institutes of Surgery by our contemporary of Dublin, will form a pendent to those we have alluded to.

*Case* 1.—It was that of a man, aged fifty, of intemperate habits, whose left shoulder was dislocated by the fall of a heavy chest. The first practitioner mistook the accident for fracture, and mistreated it accordingly, and the earliest attempt at reduction was near three weeks after. The most forcible, but otherwise judicious, means were tried without effect; and the man still anxious to have a useful limb, no matter at what expense of suffering, came to Philadelphia to consult Dr. Humphrey, by whom he was referred to Dr. Gibson, two months after the receipt of the injury.

By the abstraction of a large quantity of blood, the persevering use of the pulleys, &c. for about an hour and a half, till the patient was quite exhausted, the reduction was effected.

About an hour after

"There was a general swelling about the deltoid and pectoral muscles, which was noticed both by Dr. Humphrey and myself; but supposing it to be an approach to inflammation, a consequence to be expected after the efforts made to restore the head of the bone, nothing was apprehended from it. The swelling, however, increased very slowly for several hours; and although remarked by the house pupils and attendants, did not excite any alarm, inasmuch as the patient complained of little pain, and conversed cheerfully with some of his friends during the greater part of the afternoon. About six o'clock in the evening, Dr. Brinton, one of the house-pupils, visited him, and hearing that he had a short time before turned over in bed in order to sleep, and struck with the unusually palid appearance of his face, was induced to suspect that some unfavourable change had taken place. These suspicions were confirmed, for, upon examination, the pulse was found scarcely perceptible, and the whole system so much sunk as to render recovery impossible. Leaving Dr. Hopkinson in charge of the patient, Dr. Brinton immediately repaired to my house, and informed me of his condition. Before I could reach him, however, he expired. The appearance of the shoulder and adjacent parts soon explained, it seemed to me, the nature of the case; for the pectoral muscle was considerably elevated, and the skin for some distance about the chest and shoulder discoloured and ecchymosed, showing, in all probability, that some large artery or vein had been torn across, during the efforts to reduce the luxation. To determine this point with accuracy, I obtained the consent of the patient's friends to examine the body; and at ten o'clock next morning, the dissection was made by Drs. Horner and Lawrence, in presence of Drs. Humphrey, Jackson, the house-pupils, and myself.

Under the axillary vein, as it passes near the glenoid cavity, a large mass of coagulated blood was observed; and on clearing this away, *the axillary artery was seen protruding with its mouth open, having beeu torn directly across*, and separated from its connexions. Upon further examination it was discovered that the head of the bone, at the time of the luxation, had been carried downwards into the axilla, about an inch and a half below the glenoid cavity, where it formed a white ligamentous, cup-like, socket in the subscapularis muscle, and pressing upon the axillary artery, produced such a degree of inflammation as gave rise to a copious effusion of coagulable lymph, which united the artery completely for some distance to the capsule of the joint, where it surrounded the neck of the bone. The lower part of the capsule was torn and separated from the neck of the humerus; the upper part remained entire, and was very much thickened. The head of the bone filled completely the old sockets or glenoid cavity. Beneath the deltoid muscle there was a large hollow filled with blood, and the whole arm, as far as the elbow, had been extensively injected with the same fluid. The os humeri was carefully dissected from the condyles to its head, and the periosteum entirely scraped off, without showing the slightest vestige of a fracture. The long tendon of the biceps was found considerably elongated, but not ruptured."

*Case* 2. It was that of John Layton, ætat. thirty-five, a muscular, athletic man, about six feet high, accustomed to the use of a pint of spirits daily. The left os humeri had been dislocated nine weeks before, by a violent fall on the shoulder. Before coming under Dr. Gibson's care, four different attempts had been made at reduction, much force used, and once the body suspended upon the axilla over the top of a door. For nine days he was kept on low diet, lost ten ounces of blood daily, gentle motion was given to the arm in various directions every day, and careful friction with oils applied to the shoulder. After this the reduction was attempted.

The plan was pursued unremittingly until the 15th of March, on which day it was determined to attempt to restore the bone to its natural situation. After giving a description of the attempts at reduction, he says:

"Our efforts, notwithstanding, were continued in the most gentle and cautious way possible, sometimes by the pulleys, sometimes by the heel in the axilla, when we had the satisfaction at last, to find the head of the bone gradually approach, and finally enter, with an audible snap, the glenoid cavity; but not until the lapse of an hour and three-quarters from the commencement of the operation.

The patient, after being put to bed, complained chiefly of weakness, of numbness throughout the arm, of slight excoriation of the arm-pit, and bend of the arm. In other respects, he said, he was comfortable, and slept during the night without an opiate.

March 16. I saw him at eight in the morning, and observed a general swelling over the deltoid and part of the pectoralis major muscles, but not more than could have been expected under similar circumstances. Upon pressing, however, upon the most prominent part of the swelling, I was surprised to find a distinct pulsation of an aneurismal character. During the day both the swelling and pulsation slowly increased, and satisfied me that an aneurism existed in the axillary artery. Dr. Barton saw the patient in the afternoon and drew the same conclusion."

It was determined in consultation to tie the subclavian artery, which was done the second day after the operation. On the seventh day after the patient died, gangrene of the arm having supervened. The artery was found closely adherent to the head of the bone and capsular ligament by dense cellular or ligamentous substance, so that it appeared impossible to effect the reduction of the head of the bone and not cause the rupture of the vessel. Such had taken place; the inner and middle coats of the artery where it adhered to the bone had been ruptured, and the external expanded into a true aneurismal sac; this had burst posteriorly, and formed a diffuse aneurism. The aneurismal walls were so well defined and compact, that Dr. Gibson is inclined to think the aneurism existed prior to the last attempt at reduction, and had been then burst open.

Dr. Gibson concludes, and we think him right, that where no adhesion exists between the artery and surrounding parts, the operation may be done with safety; that, on the contrary, when adhesion does exist (and of this we have no means to judge,) rupture of the vessel must be an inevitable consequence, whether the reduction be effected by force or by the most gentle means.

He is disposed to condemn, in the most unqualified terms, all attempts at restoration of *ancient* luxations of the humerus and other bones, except in cases where the patient is remarkably thin and debilitated, and where there has been little or no inflammation at the time or subsequent to the displacement.

CLINICAL REPORT. By ALEXANDER WATSON, Surgeon to the Royal Infirmary of Edinburgh.*

Mr. Watson's main object is to discountenance a rage for operating, and to shew that great operations are often a great evil.

"Fortunately, however," he says, "the cultivation of morbid anatomy and pathology has done much to lessen, to a very surprising extent,† the number of really necessary operations, not only by pointing out more rational means of curing diseases, but also by showing the impropriety of resorting to operations, in many cases where the general system is either the source of the local disease, or is so much affected that success is hopeless; and, consequently, that operation, in such cases, is cruel as well as absurd. The increased attention, also, which is devoted to the study of anatomy and surgery, is rendering every surgeon quite competent to the performance of all the ordinary operations of surgery, both from their greater simplification and being better understood.

In consequence of the undue importance attached to operations, the principles deduced from experience, by the highest authorities in the profession, as to the propriety or impropriety of operating in particular cases, are either misunderstood, or are disregarded for the vanity of a display in flourishing the mutilating knife upon a fellow-creature, even in desperate or hopeless cases. In some such cases the chief argument I have heard used for operating was, 'to give the patient a chance,' as if the shock from the amputation of a limb was calculated to revive the expiring embers of life! In others the ingenuity of the surgeon seemed put upon the rack to create reasons 'to justify the operation,' which, if a measure really necessary to preserve life, required no other justification than that there are good grounds for expecting success.

But when a surgeon either resolves to perform a capital operation, which endangers the life of the patient, upon a chance of success, implying that success, though possible, by some fortuitous or miraculous escape that may occur, is very improbable; or finds himself under the necessity of ransacking his ingenuity to contrive arguments to justify his proceedings, he may well pause and inquire what is the rule of duty in such a case. Should he not inquire what are the principles of the profession, which reason and experience have established as applicable to such cases? what the duty of one man to another? whether or not he would sanction the operation if he was himself the patient, or if the case was that of his own child?

In cases of injury or disease capable of being cured only by a capital operation, before resorting to this measure, the following questions should always be satisfactorily answered; 1st, Is the operation necessary to save the life of the patient? 2nd, Is there a fair prospect of success? Unless the case stands this simple test, the performance of the operation would be decidedly improper.

In proof of the correctness of these statements, by acting upon the principles here described, I have, during the last four years that I have been connected with the Infirmary, performed only 16 amputations of the extremities, 13 having been in the house and 3 in private. These operations were performed partly on account of severe injuries, and partly for diseases of the bones and joints. They consisted of amputations above the knee, 7; of the leg, 4; of shoulder joint, 2; of arm above elbow, 3; making altogether, 16. Of these, 15 recovered and only one died. But had these principles been deviated from, and had other

---

* Edinburgh Journal, July 1839.

† "Mr. Lawrence of St. Bartholomew's Hospital says, in his Lectures, to an extent of 'one-half or two-thirds within the last twenty-five years.'"

cases of patients in a dying state been operated on, to give them 'a chance,' my success would have been very different."

We extract an observation or two, on the subject of injuries of the head. Speaking of *concussion*, Mr. Watson remarks :—

That the disorder or lesion produced by concussion is chiefly situated in the hemispheres of the cerebrum, seems highly probable, from the state of the patients almost completely resembling that of animals from whose heads these hemispheres have been removed. According to the experiments of physiologists, (see Müller,) animals in this state could be kept alive for a great length of time; they lost their intellectual faculties, as also their sight and hearing; but they retained muscular irritability, and those functions which are, in a great measure, independent of the hemispheres of the cerebrum. Hence they swallowed food, and the animal functions were carried on, but they seemed as if always asleep and disliked to be disturbed. These functions, though (for their perfect state) dependent on the integrity of the whole nervous system, are carried on chiefly by the influence of the *medulla oblongata*, the spinal cord, and sympathetic nerve; while the disturbed and diminished power of the cerebrum tends to lessen, and in some cases to annihilate their power.

He makes some observations on compression of the brain arising from extravasation of blood. We extract the following :—

To ascertain the situation of blood effused within the cavity of the cranium with precision, is a problem beset with many difficulties. There are three situations in which it may occur, namely, between the skull and the *dura mater*, between the *dura mater* and surface of the brain, or into the interior of the brain. But the effusion does not always take place at the situation of an external injury, as is remarkable in cases 4, 5, and 6. On the contrary, it often takes place in the opposite side of the cranium, and yet the symptoms are precisely similar.

It is of importance to observe, however, that when the effusion is at the base of the cranium, compressing the *medulla oblongata*, death is generally immediate; whereas, if the blood is situated at any of the sides or upper part of the cranium, the patient, though he becomes apoplectic, may live for a considerable number of hours or even days.

Another circumstance worthy of remark, is that relating to the opinion stated by the late Mr. Abernethy and Sir B. Brodie, that blood is never poured out in such quantity between the bone and *dura mater* as to produce dangerous pressure upon the brain, 'except where the middle meningeal artery has been lacerated.'* Sir B. Brodie further states, that he had not seen a case of laceration of this vessel, without a fracture running across its bony canal. But he adds, that some cases of this artery being opened without fracture of the bone have been recorded by others.

Here, then, we have evidence, at least, of the frequent occurrence of rupture of the meningeal artery, when the bone, along which it is situated, is fractured.

In cases, therefore, accompanied with symptoms of compression of the brain from effusion of blood within the cranium, when death does not take place suddenly, when there is an injury in the temporo-parietal region, but more especially when there is a fracture of the skull in this situation, and bleeding from the ear the *probability* of this being the seat of the effused blood amounts almost to *certainty*.

---

* Medico-Chirurgical Transactions, v.—xiv.

